# Adequate water supply enhances seedling growth and metabolism in *Festuca kryloviana*: insights from physiological and transcriptomic analys

**DOI:** 10.1186/s12870-024-05353-5

**Published:** 2024-07-26

**Authors:** Zhenghai Shi, Guoling Liang, Sida Li, Wenhui Liu

**Affiliations:** 1 Key Laboratory of Superior Forage Germplasm in the Qinghai-Tibetan plateau, Qinghai Academy of Animal Science and Veterinary Medicine, Qinghai University, Xining, 810000 Qinghai China; 2 Laboratory for Research and Utilization of Qinghai Tibet Plateau Germplasm Resources, Chengbei District, Xining City, Qinghai Province China

**Keywords:** Water management, Qinghai Lake region, Seedling development, Sugar metabolism, Antioxidant system

## Abstract

**Background:**

*Festuca kryloviana* is a significant native grass species in the Qinghai Lake region, and its low emergence rate is a primary factor limiting the successful establishment of cultivated grasslands. The region’s arid and low-rainfall climate characteristics result in reduced soil moisture content at the surface. Despite the recognized impact of water availability on plant growth, the specific role of moisture in seedling development remains not fully elucidated. This study aims to investigate the germination rate and seedling growth velocity of *F. kryloviana* seeds under varying moisture conditions, and to integrate physiological and transcriptomic analyses of seedlings under these conditions to reveal the mechanisms by which water influences seedling development.

**Results:**

The emergence rate of *F. kryloviana* seedlings exhibited an initial increase followed by a decrease with increasing moisture content. The highest emergence rate, reaching 75%, was observed under 20% soil moisture conditions. By the eighth day of the experiment, the lengths of the plumules and radicles under the optimal emergence rate (full water, FW) were 21.82% and 10.87% longer, respectively, than those under closely matching the soil moisture content during the background survey (stress water, SW). The differential development of seedlings under varying moisture regimes is attributed to sugar metabolism within the seeds and the accumulation of abscisic acid (ABA). At FW conditions, enhanced sugar metabolism, which generates more energy for seedling development, is facilitated by higher activities of α-amylase, sucrose synthase, and trehalose-6-phosphate synthase compared to SW conditions. This is reflected at the transcriptomic level with upregulated expression of the α-amylase (*AMY2*) gene and trehalose-6-phosphate synthase (*TPS6*), while genes associated with ABA signaling and transduction are downregulated. Additionally, under FW conditions, the expression of genes related to the chloroplast thylakoid photosystems, such as photosystem II (PSII) and photosystem I (PSI), is upregulated, enhancing the seedlings’ light-capturing ability and photosynthetic efficiency, thereby improving their autotrophic capacity. Furthermore, FW treatment enhances the expression of the non-enzymatic antioxidant system, promoting metabolism within the seeds. In contrast, SW treatment increases the activity of the enzymatic antioxidant system, including peroxidase (POD), superoxide dismutase (SOD), and catalase (CAT), to cope with water stress.

**Conclusions:**

Our experiment systematically evaluated the impact of moisture conditions on the growth and development of *F. kryloviana* seedlings. Physiological and transcriptomic data collectively indicate that adequate water (20%) supply enhances seedling growth and development by reducing ABA levels and increasing α-amylase activity within seeds, thereby boosting sugar metabolism and promoting the growth of seedling, which in turn leads to an improved emergence rate. Considering water management in future cultivation practices may be a crucial strategy for enhancing the successful establishment of *F. kryloviana* in grassland ecosystems.

**Supplementary Information:**

The online version contains supplementary material available at 10.1186/s12870-024-05353-5.

## Background

The developmental process of seedlings is characterized by three distinct stages: imbibition, germination, and seedling elongation [1, 2], with the health status during the elongation phase being pivotal for successful emergence. Research has established that seedling development is influenced not only by seed size and planting depth but also significantly by soil moisture levels [3]. Water is essential for maintaining plant turgor pressure; however, a deficiency leads to a loss of this pressure due to insufficient water for basic functions, thereby inducing water stress [4]. Plants have evolved complex mechanisms to cope with environmental stress, including the perception of stress signals, signal transduction pathways, and the activation of stress-responsive genes and metabolites [5, 6]. Under water stress conditions, plants produce and accumulate high levels of reactive oxygen species (ROS), leading to physiological phenomena such as lipid peroxidation, membrane degradation, and DNA modification [7, 8]. Malondialdehyde (MDA), a product of cellular membrane lipid peroxidation, serves as an indicator of plant damage under stress [9]. To mitigate the damage caused by the accumulation of ROS, plants activate the expression of enzymatic and non-enzymatic antioxidant systems to scavenge ROS. Signal perception and transduction through pathways involving mitogen-activated protein kinase (MAPK), abscisic acid (ABA), reactive oxygen (ROS), and calcium-dependent protein kinases (Ca^2+^/CAM) [10]ultimately lead to the activation of transcription factors (TFs) that induce enzymes such as superoxide dismutase (SOD) and peroxidase (POD), which are involved in the suppression of oxidative stress [11–13]. SOD, POD, and catalase (CAT) are integral components of the plant’s non-enzymatic antioxidant system [14]. Notably, POD is responsible for the removal of O_2_ and H_2_O_2_ rapidly converted by the SOD-catalyzed reaction [8].

During the early stages of seedling growth, energy and non-photosynthetic carbon sources for growth and development are primarily derived from the metabolism of reserves stored within the seed [15]. Starch, proteins, lipids, nucleic acids, and mineral complexes stored in the endosperm are hydrolyzed by corresponding enzymes into smaller molecules such as sugars, reduced nitrogen, and other soluble nutrients, which are then transported to the growing tissues via endocytosis or transport proteins to support seedling growth [16]. Among all hydrolytic enzymes, α-amylase is the most abundant and plays a crucial role in controlling the rate of starch degradation and mobilization [17]. Gibberellins (GAs) are the primary hormones that initiate metabolism within the endosperm [17, 18]. Studies have shown that GA actively promotes the synthesis of α-amylase during the early growth of plant seedlings, influencing processes such as seed germination, root growth, and leaf expansion [19, 20]. ABA, a key antagonistic regulator of GA, has been extensively studied. It is known that ABA and sugar accumulation signals interact to inhibit the expression of α-amylase, maintaining the seed embryo in a dormant state and thus inhibiting seedling development [21].

*Festuca kryloviana* is a native grass species in the Qinghai Lake region, and the utilization of such local grass species is essential for the restoration of local ecosystems. However, uneven or low emergence rates have been observed in grassland establishment, making the improvement of emergence rates a crucial direction for the successful cultivation of artificial grasslands of *F. kryloviana*. Based on findings from other plants, the health of the elongation stage is key to successful emergence, and water deficiency inhibits seedling development [3, 4, 13, 22]. Aridity is a primary climatic feature of the Qinghai-Tibet Plateau, and during the *F. kryloviana* planting season, there is virtually no rainfall, with high evaporation rates potentially further reducing soil surface moisture content. Despite this, there have been no studies on the effects of moisture conditions on the growth of *F. kryloviana* seedlings. Therefore, we conducted gradient experiments with different moisture levels to observe the seedlings’ response to water. We aimed to elucidate the mechanisms by which moisture conditions affect seedling growth by measuring sugar metabolism and antioxidant indicators, combined with gene expression analysis. We hope that our findings will provide a reference for the water management of seedlings in artificial grasslands in the Qinghai Lake region and similar areas.

## Results

### Soil background investigation

In our investigation of the experimental site and the surrounding soil background, a distinct trend in soil moisture content with increasing soil depth was observed, characterized by an initial increase followed by a subsequent stabilization (Fig. S1). The average soil moisture content in the 0–10 cm soil layer was 11.61%, representing a decrease of 23.77% compared to the moisture content in the 20–30 cm soil layer.

### Seed germination rate and seedling emergence rate

**Fig. 1:**
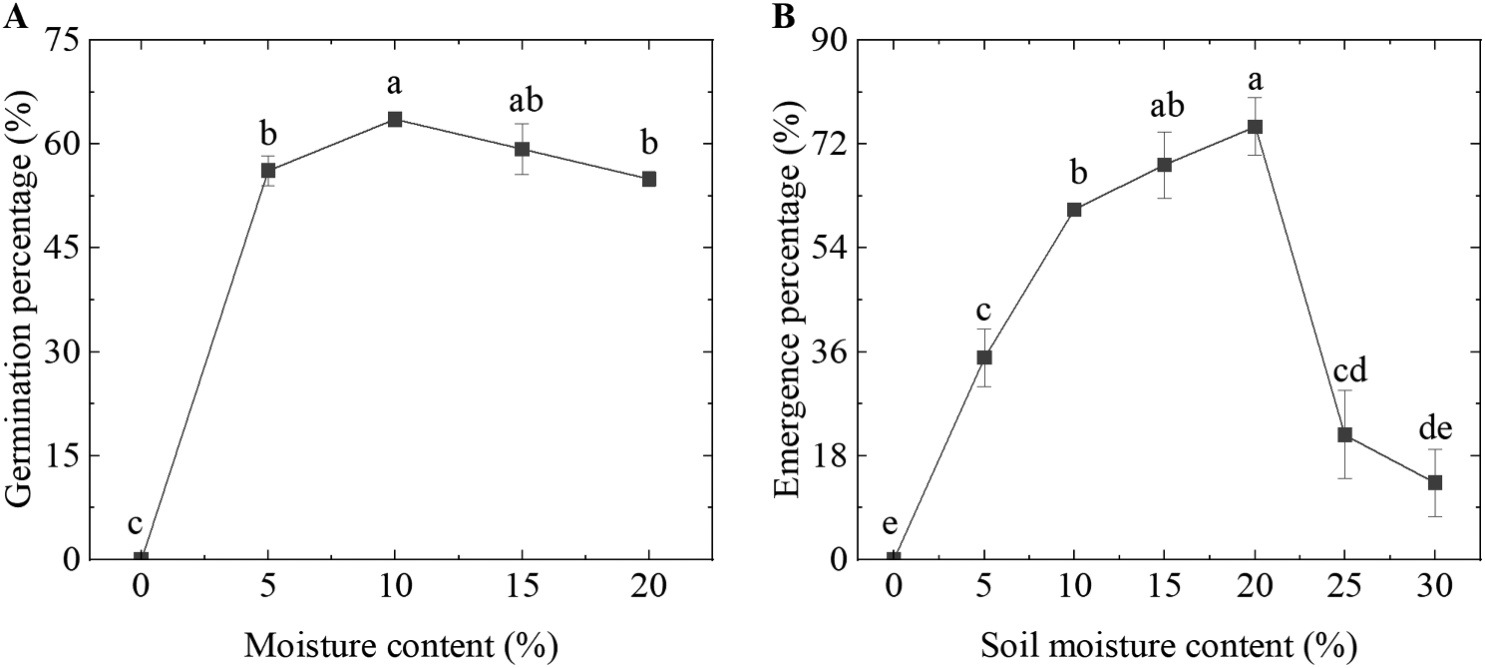
Impact of moisture content on the germination and emergence rate. Germination rate of germination box **(A)** and emergence rate of soil environment **(B)**. Water accumulation in the germination box and soil environment began at 20% and 25% moisture content, respectively

In our experiments, a non-linear trend in seed germination rate with increasing moisture content was observed, characterized by an initial increase followed by a subsequent decrease (Fig. 1A). Seeds subjected to the treatment without added water showed no germination. The highest germination rate reached 63% and was observed at a moisture content of 10%. As moisture content continued to increase, water accumulation in the germination box resulted in a reduction in seed germination rates. To test whether moisture content affects seedling emergence, our soil moisture content experiments revealed that the maximum emergence rate of seeds occurred at a soil moisture content of 20% and reached 75% (Fig. 1B). After soil wetting, there was a significant decrease in emergence rates, with an 82.66% decrease in soil moisture content observed at 30% compared to 20%.

### Radicle and plumule growth

Our experimental results indicated that the growth of radicles and plumules were influenced by moisture, time, and their interaction (*P* < 0.01, Table S1). During the 8–20 days of our experiment, the length of radicles under the FW treatment was 10.87-29.08% higher than under the SW treatment (Table 1). After 22 days, there was no significant difference in radicle length between the two moisture treatments (*P* < 0.05). The length of plumules under both moisture treatments increased initially and then plateaued, but the FW treatment was more conducive to seedling growth. Eight days after the experiment began, the length of plumules under the FW treatment was more than 21.82% higher than under the SW treatment. At 14 and 18 days into the experiment, the lengths of plumules under the FW and SW treatments were 46.40 mm and 37.90 mm, respectively.

**Table 1:** Growth characteristics of seedlings under different moisture conditions. “/“ indicates non-statistical data. Different uppercase letters indicate significant differences between different moisture treatments at the same time point, and different lowercase letters indicate significant differences between different time points under the same moisture treatment

Traits	Days (d)	FW (mm)	SW (mm)
Radicle length	2	/	/
	4	3.97 ± 0.68Ah	3.13 ± 0.44Bi
	6	9.31 ± 0.38Bg	11.22 ± 0.36Ah
	8	18.45 ± 0.99Af	16.64 ± 0.49Bg
	10	24.22 ± 1.84Ae	21.23 ± 1.86Bf
	12	28.34 ± 1.74Ade	23.65 ± 1.40Bef
	14	30.81 ± 2.69Acd	24.97 ± 2.34Bde
	16	35.28 ± 1.75Abc	27.61 ± 1.76Bd
	18	36.60 ± 2.29Aab	28.35 ± 1.63Bd
	20	36.51 ± 2.09Aab	32.30 ± 1.9B8c
	22	38.86 ± 3.64Aab	36.04 ± 1.66Ab
	24	41.13 ± 4.63Aa	39.74 ± 2.75Aa
Plumule Length	2	/	/
	4	9.02 ± 1.67Af	7.54 ± 0.93Ah
	6	19.28 ± 1.26Ae	17.52 ± 1.15Ag
	8	26.42 ± 2.83Ad	21.31 ± 0.58Bf
	10	37.23 ± 1.67Ac	26.81 ± 0.69Be
	12	42.50 ± 2.22Abc	30.17 ± 1.34Bde
	14	46.40 ± 5.64Aab	32.94 ± 2.14Bcd
	16	48.54 ± 2.39Aab	34.79 ± 1.53Bbc
	18	48.99 ± 1.53Aa	37.90 ± 1.53Bab
	20	49.44 ± 3.18Aa	38.53 ± 3.33Ba
	22	49.13 ± 1.45Aa	39.84 ± 0.57Ba
	24	49.44 ± 4.47Aa	40.58 ± 2.02Ba

### Sugar metabolism and related enzyme activities

**Fig. 2:**
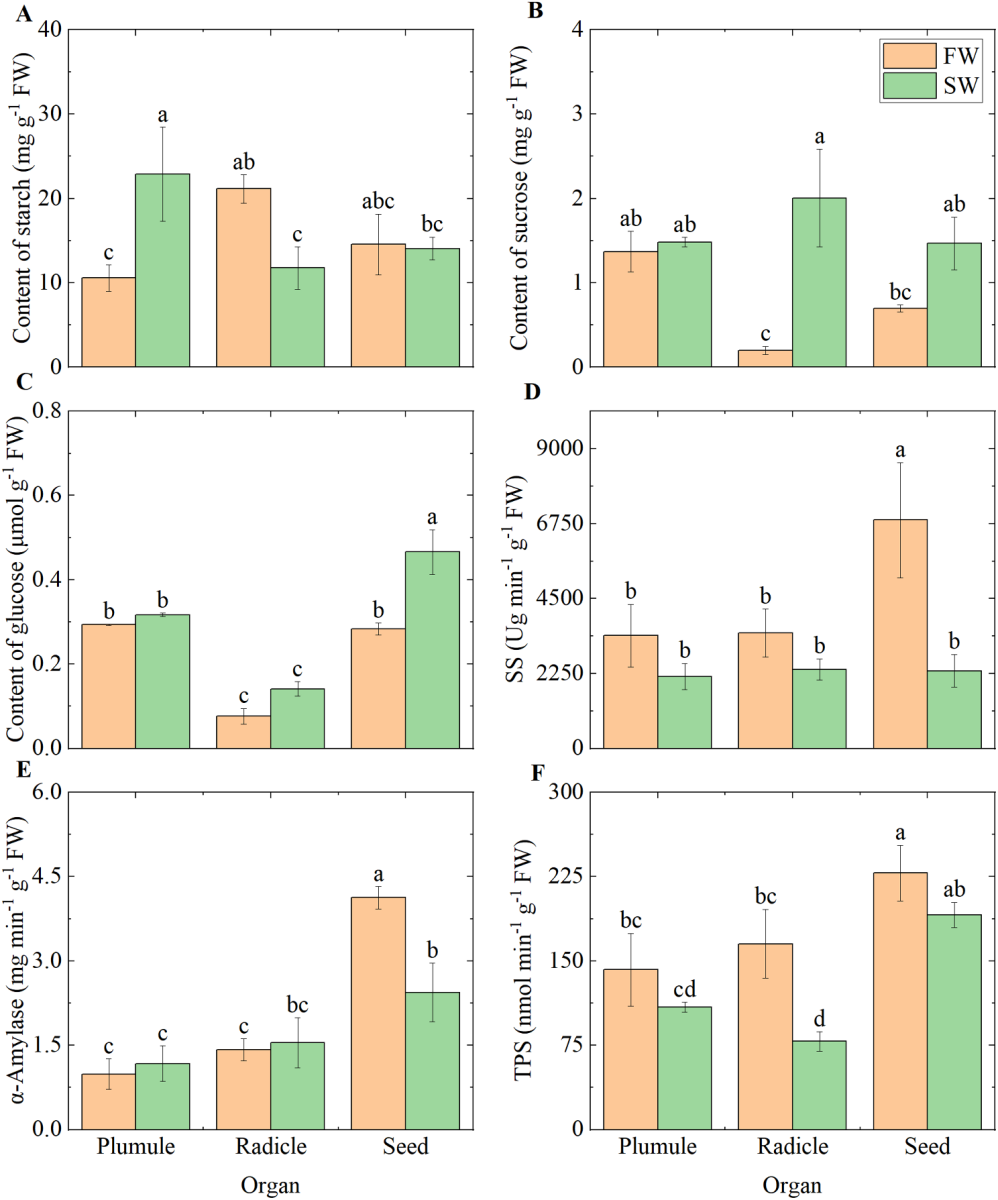
Sugar metabolism and related enzyme activities in different organs under varying moisture conditions. **(A)** Starch; **(B)** Sucrose; **(C)** Glucose; **(D)** Sucrose synthase; **(E)** α-amylase; **(F)** Trehalose-6-phosphate synthase. SS indicates sucrose synthase; TPS indicates trehalose-6-phosphate synthase

We determined in our study that the starch content in the plumules was 116.48% higher under the SW treatment compared to the FW treatment (Fig. 2A). The starch content in the radicles and the activity of TPS under the SW treatment were 44.47% and 52.66% lower than under the FW treatment, while sucrose was 905.61% higher under the SW treatment (Fig. 2B and F). In the seeds, SS and α-amylase activities under the SW treatment were 65.93% and 40.87% lower than under the FW treatment, while sucrose and glucose were 157.30% and 64.58% higher under the SW treatment, respectively (Fig. 2B-E). Additionally, we found that seeds under the FW treatment had higher α-amylase activity, SS activity, and TPS activity in both radicles and plumules.

### Antioxidant characteristics

**Fig. 3:**
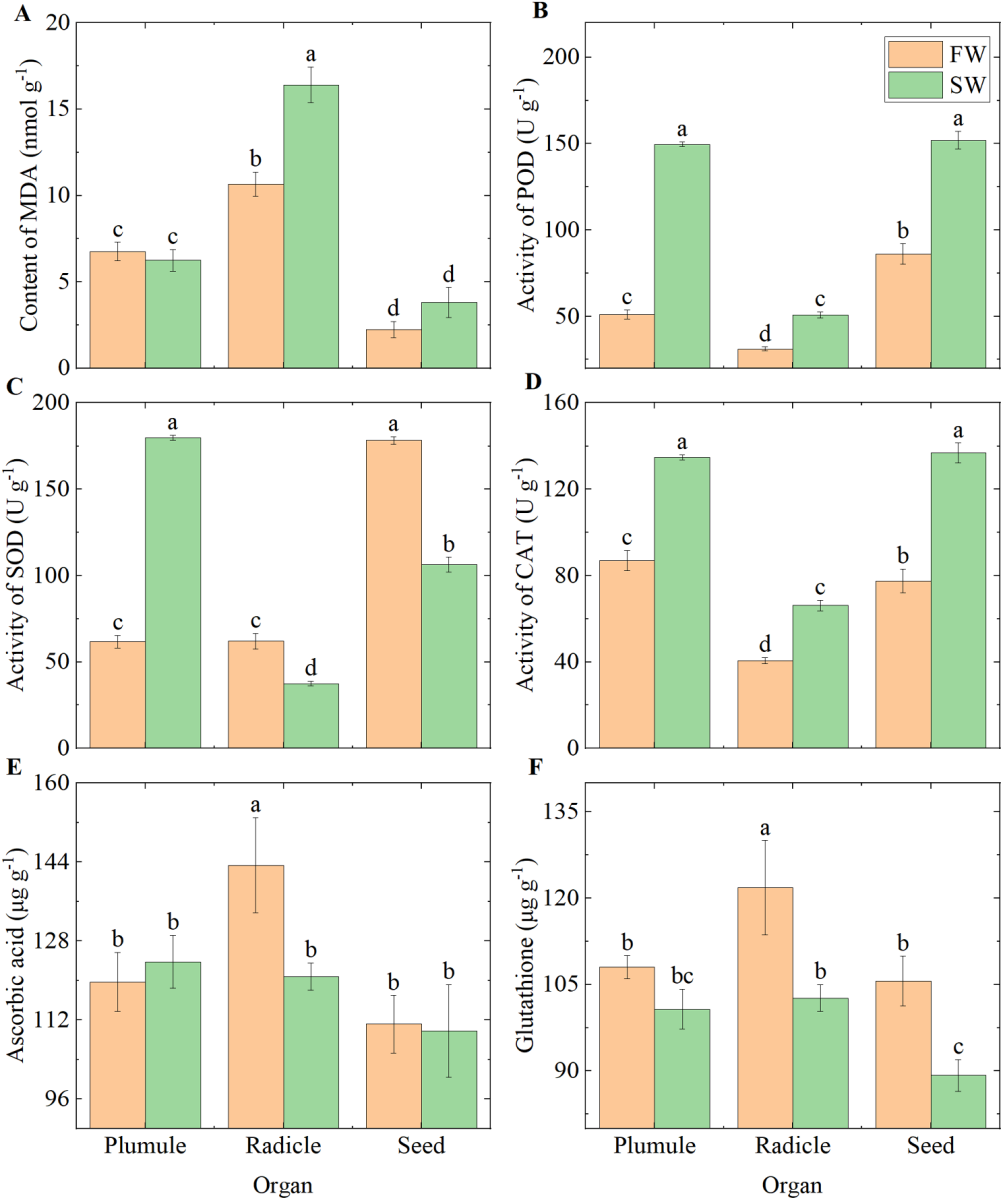
Antioxidant indices in various organs under different moisture conditions. **(A)** MDA; **(B)** POD; **(C)** SOD; **(D)** CAT; **(E)** Ascorbate; **(F)** Glutathione

Our antioxidant assays revealed that the content of MDA in radicles under the FW treatment was 35.04% lower compared to the SW treatment, while the content of ascorbate (AsA) was 18.68% higher under the FW treatment (Fig. 3A and E). The activity of POD in plumules, radicles, and seeds under the FW treatment was 65.85%, 38.63%, and 43.31% lower than under the SW treatment, respectively. CAT showed a similar trend to POD (Fig. 3B and D). In radicles and seeds, glutathione (GsH) content showed a similar pattern to SOD (Fig. 3C and F). The SOD levels were 65.59% and 67.48% higher in the FW treatment than in the SW treatment, respectively.

### Hormone content

**Fig. 4:**
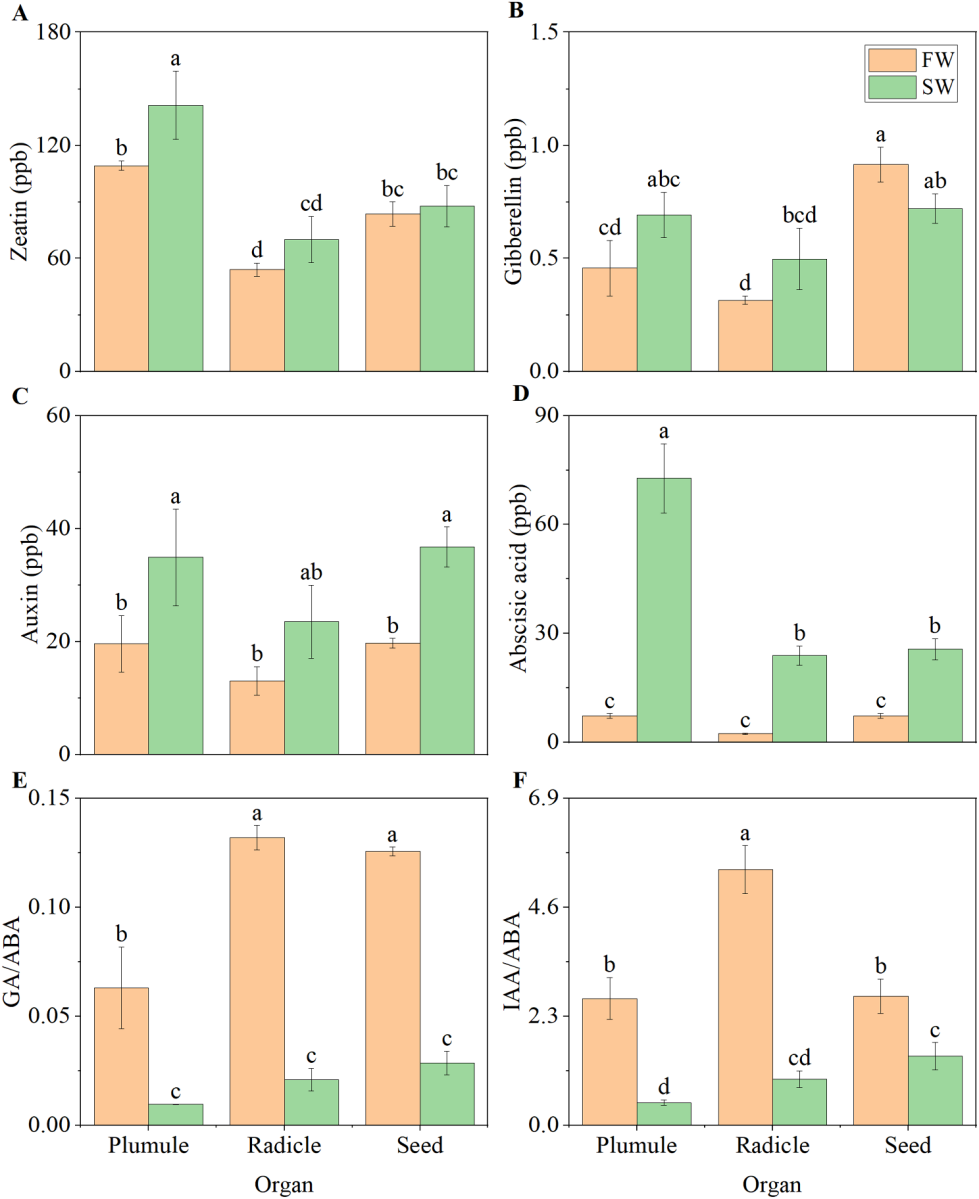
Hormone contents in various organs under different moisture conditions. **(A)** ZR; **(B)** GAs; **(C)** IAA; **(D)** ABA; **(E)** GA/ABA; **(F)** IAA/ABA

We found that the content of zeatin (ZR) and IAA in plumules under the FW treatment was 22.66% and 43.74% lower than under the SW treatment (Fig. 4A and C), respectively. The content of ABA under the FW treatment was 89.99% lower than under the SW treatment (Fig. 4D). The GA/ABA and IAA/ABA ratios under the FW treatment were 6.64 and 5.63 times those under the SW treatment (Fig. 4E and F), respectively. In radicles, the content of ABA under the FW treatment was 89.97% lower than under the SW treatment, and the GA/ABA and IAA/ABA ratios under the FW treatment were 6.32 and 5.55 times those under the SW treatment (Fig. 4D-F), respectively. In seeds, the content of IAA and ABA under the FW treatment was 46.39% and 71.52% lower than under the SW treatment (Fig. 4C and D), respectively. The GA/ABA and IAA/ABA ratios under the FW treatment were 4.39 and 1.87 times those under the SW treatment, respectively. Further analysis of the physiological indices in plumules, radicles, and seeds revealed that, compared to GA, ABA and IAA had stronger correlations with antioxidant and sugar metabolism indices, particularly in seeds (Fig. S2).

### Transcriptome data annotation

**Fig. 5:**
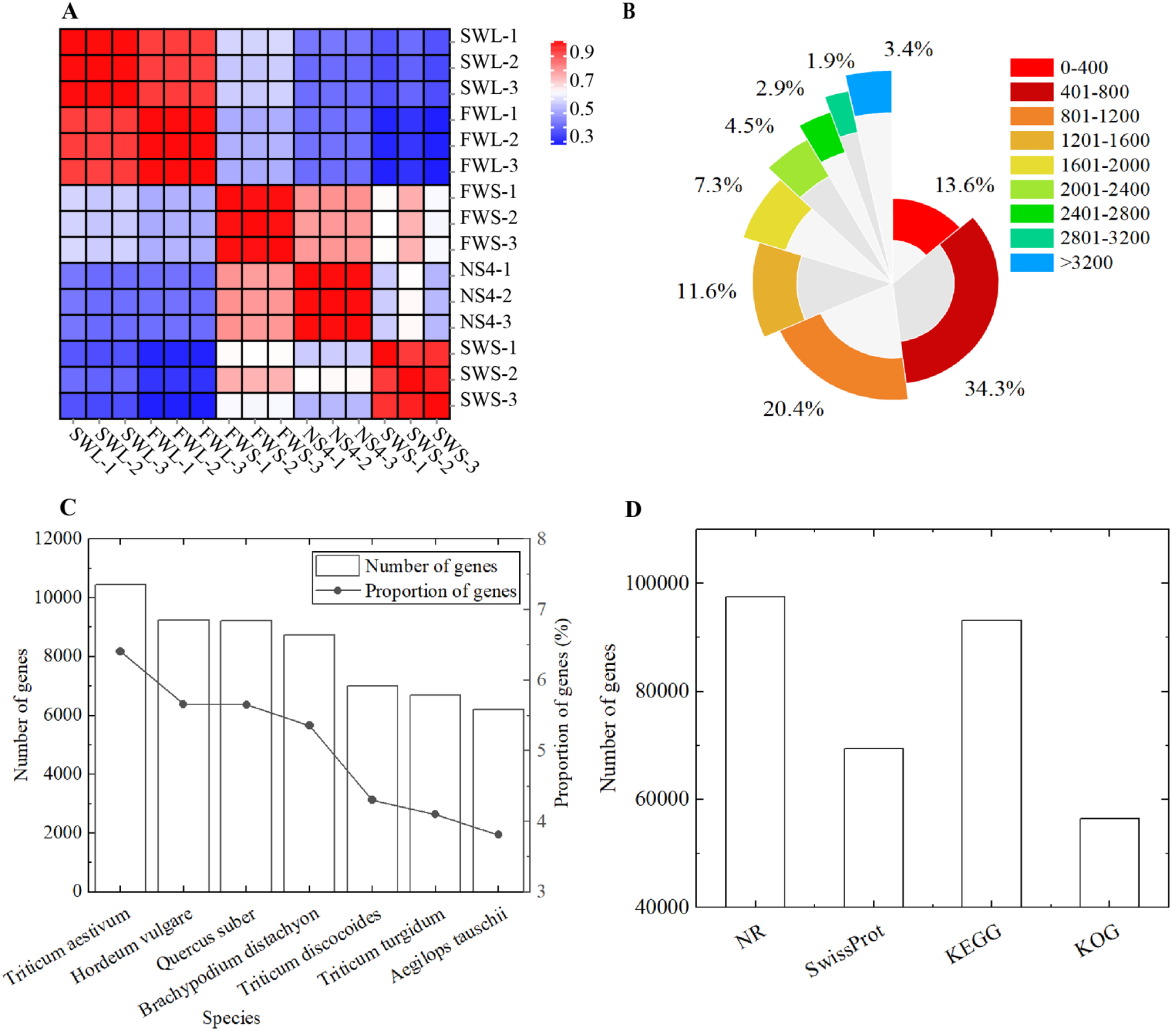
Functional annotation of the transcriptome data for *F. kryloviana* from the Qinghai Lake Region. **(A)** Assessment of biological replicates for gene expression consistency; **(B)** Distribution characteristics of gene lengths; **(C)** Species annotation of assembled genes; **(D)** Functional annotation of assembled genes

We observed a strong correlation in gene expression between biological replicates of each sample, with correlation coefficients all greater than 0.95 (*P* < 0.05, Fig. 5A). Using the Trinity software, we assembled the data after removing low-quality reads and adapter sequences, resulting in 163,114 unigenes. The length distribution of the assembled genes is as follows (Fig. 5B): 12.69% of the genes were longer than 2000 bp, the average gene length was 1106 bp, and the N50 length was 1461 bp. Based on the functional annotation results of the NR database, the proportions of different species in the notes of genes were calculated, with 11,043 (6.43%) genes aligned to Triticum aestivum (Fig. 5C). The assembled genes were functionally annotated using four databases, with 97,466 (59.75%), 69,440 (42.57%), 93,096 (57.07%), and 56,432 (34.60%) genes annotated in the NR, SwissProt, KEGG, and COG/KOG databases, respectively. However, 63,749 (39.08%) genes remained unannotated in the four databases (Fig. 5D).

### Differential gene expression in seedling organs: GO enrichment and KEGG pathway analysis

**Fig. 6:**
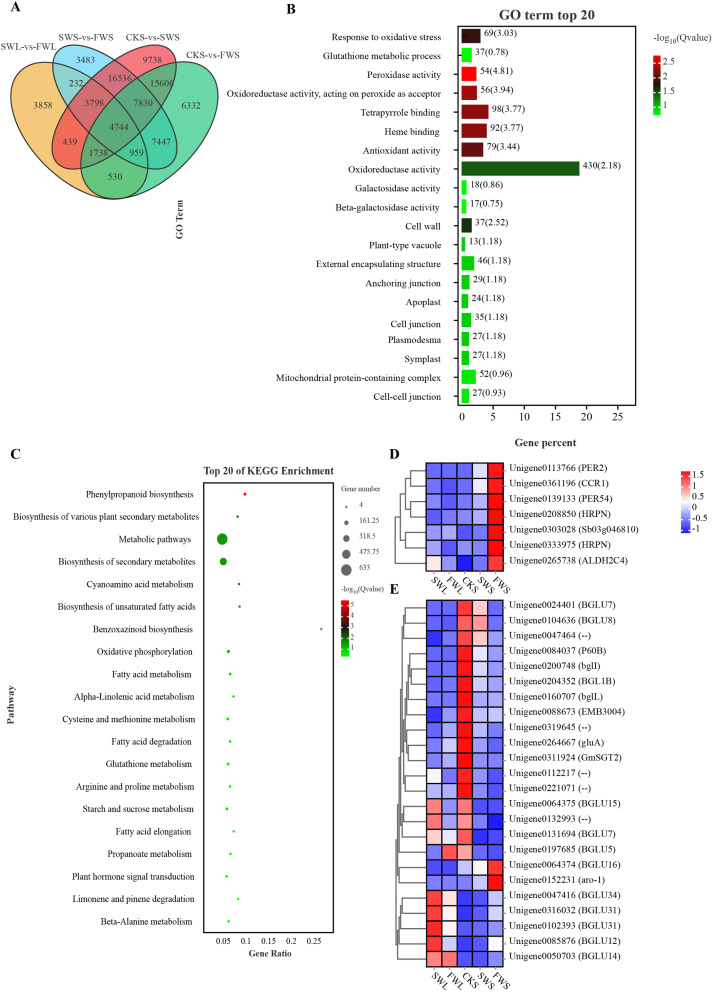
Expression of differentially expressed genes in plumules across distinct water treatments. **(A)** Enrichment of Gene Ontology (GO) terms; **(B)** Enrichment of Kyoto Encyclopedia of Genes and Genomes (KEGG) pathways; **(C)** Expression of differentially expressed genes involved in the photosynthesis-antenna proteins process; **(D)** Expression of major differentially expressed genes within the Light-harvesting Chlorophyll Protein Complex (LHC) Lhca; **(E)** Expression of major differentially expressed genes within the Light-harvesting Chlorophyll Protein Complex (LHC) Lhcb. SWL and FWL represent plumules grown for eight days under SW treatment and FW treatment, respectively

Analysis of differential gene expression within the plumules revealed that under full water (FW) conditions, 5,197 genes were upregulated and 11,101 genes were downregulated compared to stress water (SW) conditions (Fig. S3). We conducted Gene Ontology (GO) enrichment analysis to assess the functions of the differentially expressed genes (DEGs) during the growth process of the plumules, comparing the top 20 significantly enriched GO terms related to cellular components (CC), molecular functions (MF), and biological processes (BP). The SW vs. FW comparison highlighted the enrichment of terms associated with nitrogen compound biosynthetic processes, chloroplast thylakoid membrane, plastid thylakoid membrane, structural constituent of ribosome, and thylakoid (Fig. 6A). This suggests that water conditions influence the development of chloroplast thylakoid membranes and nitrogen compound synthesis processes in the seedling plumules. Further Kyoto Encyclopedia of Genes and Genomes (KEGG) pathway analysis indicated that pathways such as photosynthesis - antenna proteins, photosynthesis, ribosome, metabolic pathways, and carbon fixation in photosynthetic organisms were the most significantly enriched (Fig. 6B). Notably, among the 106 genes involved in the processes of the light-harvesting chlorophyll protein complex (LHC) of photosystem II and photosystem I, and the photosynthetic electron transport process, 11 genes showed expression fold changes up to 15 times higher under FW conditions (Fig. 6C, D, and E). Additionally, 15 out of 73 genes involved in photosystem II, photosystem I, and photosynthetic electron transport processes were significantly upregulated under FW treatment (Fig. S4).

Upon comparison, it was observed that 15,644 genes were significantly upregulated in seeds subjected to full water (FW) treatment as compared to stress water (SW) treatment. Gene Ontology (GO) analysis revealed that in the comparison between SWS and FWS, terms such as ‘ribosome,’ ‘structural molecule activity,’ ‘organonitrogen compound biosynthetic process,’ and ‘amide biosynthetic process’ were predominantly enriched (Fig. S5A). Kyoto Encyclopedia of Genes and Genomes (KEGG) pathway analysis identified significant enrichment in pathways including ‘ribosome,’ ‘valine, leucine and isoleucine degradation,’ ‘peroxisome,’ ‘tryptophan metabolism,’ and the ‘Citrate cycle (TCA cycle)’ (Fig. S5B). These results highlight the different gene expression in various organs. with a predominant focus on energy metabolism and antioxidant metabolism within the seeds.

### Gene-specific expression analysis in seeds with adequate water supply

Comparative analysis of gene expression at the just-germinated of seeds (CKS) revealed 45,454 and 33,424 upregulated genes for SW and FW treatments, respectively. Venn diagram analysis revealed that there were 6332 genes specifically expressed in the CKS vs. FWS group. GO analysis indicated that peroxidase activity, oxidoreductase activity acting on peroxide as acceptor, tetrapyrrole binding, heme binding, antioxidant activity, and response to oxidative stress were among the main enriched terms. KEGG analysis primarily highlighted pathways such as phenylpropanoid biosynthesis, biosynthesis of various plant secondary metabolites, metabolic pathways, biosynthesis of secondary metabolites, cyanoamino acid metabolism, and biosynthesis of unsaturated fatty acids. In particular, the phenylpropanoid biosynthesis pathway, which involves the synthesis of multiple compounds, had 7 genes upregulated under the FW treatment, such as *PER2/54*, *CCR1*, and *ALDH2C4*. In the biosynthesis of various plant secondary metabolites pathway, 7 BGLU-type genes involved in the pentagelloyl glucose biosynthesis and coumarin biosynthesis processes were upregulated under the FW treatment. Additionally, the starch degradation pathway was predominantly upregulated by the *AMY2* gene, and genes *TPS6* and *TPP1*, which maintain sucrose balance, were also upregulated (Fig. S6A and B). In terms of plant hormone signaling, the number of active genes related to the synthesis and transduction of auxins and ABA was significantly higher than those related to GAs (Fig. S6C and D).

### Validation of RNA-Seq by qRT-PCR analysis

We conducted quantitative real-time polymerase chain reaction (qRT-PCR) analysis on 14 differentially expressed genes to validate our transcriptomic findings. Our analysis revealed that in the plumules and seeds, 11 and 12 genes, respectively, exhibited expression trends consistent with the RNA-seq data, although the magnitude of relative expression fold changes varied (Table S3). In the plumules, *AMY2, IAA21* and *TPP1* showed no significant difference in expression by RNA-seq but were downregulated in qRT-PCR. Within the seeds, *TPP1* was upregulated in RNA-seq but downregulated in qRT-PCR. *ALDH2C4* were upregulated in RNA-seq but downregulated in qRT-PCR. The correlation between the fold changes observed in RNA-seq and qRT-PCR was found to be positive (*P* < 0.05, R² = 0.6001, Fig. S9).

## Discussion

### Water stress inhibits seedling growth and development

Water stress inhibits seedling growth and development (Fig. S7). Plants exhibit an S-shaped curve in their adaptation to environmental stress throughout their life cycle, with the seedling stage showing the least tolerance to water stress [23, 24]. Insufficient water supply leading to water stress may be a primary cause of the low emergence rate in artificially established *F. kryloviana* grasslands. Our experimental results indicate that the emergence rate of seedlings was significantly higher at soil moisture levels of 15–20% (Fig. 1). However, soil moisture content at a depth of 0–10 cm was found to be only 11.61%, resulting in a 20% reduction in emergence rate compared to conditions with 20% soil moisture (Figs. 1 and S1). Negative correlations have been observed between plant germination rate, germination potential, and radicle growth with drought stress [13, 22, 25]. Drought stress suppresses seedling development by reducing the vitality and growth rate of tomato seedlings’ root systems [25] and the photosynthetic capacity of *Vigna radiata* (L.) Wilcziek leaves [22, 26]. After the seedling establishment phase, plants gradually transition to a photosynthetically autotrophic stage, where the structural development and efficient functioning of photosynthetic organs are fundamental to seedling growth. We observed that after 8 days of experimentation, the lengths of radicles and plumules in seedlings treated with FW were 10.87% and 21.82% longer, respectively, than those in SW conditions (Table 1). A significant upregulation of genes associated with the light-harvesting chlorophyll protein complex (LHC) of the chloroplast, including Lhcal/b1, Lhca2/b2, Lhca3/b3, Lhca4/b4, Lhcb5, and Lhcb6, as well as photosystem I, photosystem II, and photosynthetic electron transport processes, was noted under FW conditions compared to SW conditions (Figs. 6 and S4). Studies have shown that upregulation of LHC-related genes in the thylakoid membrane of chloroplasts is beneficial for increasing the chlorophyll content of seedlings [13]. Similar findings have been reported in *Puccinellia tenuiflora*, where downregulation of light-harvesting complexes and Calvin cycle enzymes reduced photosynthesis [27]. Therefore, based on the observed slow growth of radicles and plumules and the downregulation of genes related to the chloroplast photosynthetic system under SW conditions, we infer that water stress inhibits the development of photosynthetic organs, thereby preventing seedlings from progressing to the photosynthetic autotrophic phase. When the limited reserves within the seed are depleted and the seedlings are still unable to enter a photosynthetic autotrophic state, growth and development cease, leading to a reduction in the emergence rate.

### Water stress induces the expression of enzymatic antioxidant systems

**Fig. 7:**
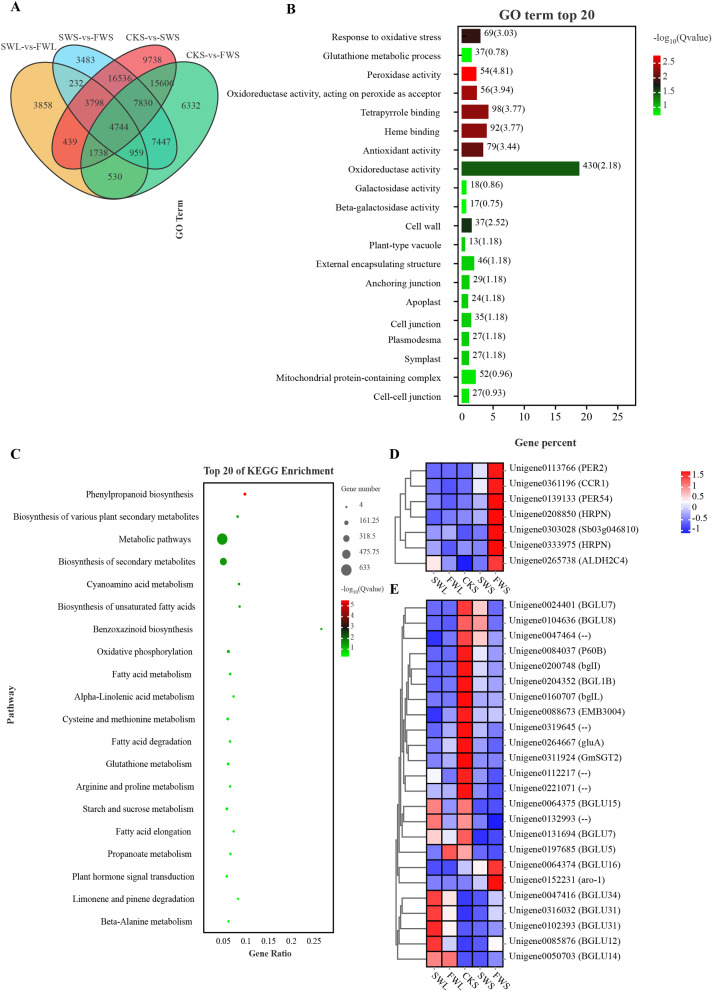
Analysis of genes specifically expressed in seeds under FW. **(A)** Specific gene expression analysis in seeds under sufficient moisture conditions; **(B)** Gene Ontology (GO) enrichment of specifically expressed genes; **(C)** Kyoto Encyclopedia of Genes and Genomes (KEGG) enrichment of specifically expressed genes; **(D)** Expression of genes within the phenylpropanoid biosynthesis pathway; **(E)** Expression of non-enzymatic antioxidant genes within secondary metabolite pathways. CKS, SWS, and FWS represent seeds in the just-germinated, stress water (SW), and full water (FW) states, respectively

Drought stress triggers the accumulation of reactive oxygen species (ROS) within plants [13]. To counteract this, plants activate the expression of their antioxidant systems to enhance their resistance. POD plays a primary role in the scavenging of ROS [28]. *Isatis indigotica* has been shown to increase the activity of antioxidant enzymes in leaves (such as SOD, POD, and CAT) to regulate the increased MDA caused by water deficiency during the vegetative growth phase [4]. mung bean, modulates oxidative stress caused by drought-induced insufficient non-photochemical quenching (NPQ) of photosystem II (PSII) by accumulating proline and enhancing POD activity [26]. Our experimental results, which show higher POD values in radicles, plumules, and seeds under SW conditions, indicate a substantial accumulation of ROS in seedlings during water deficiency (Fig. 3). The excessive accumulation of ROS can induce lipid peroxidation of cellular membranes, leading to the production of MDA [9]. Our findings of the highest MDA values and the lowest POD values in radicles, along with a 35.04% reduction in MDA content in FW-treated radicles compared to SW-treated radicles, further illustrate that seedlings are under water stress conditions under SW (Fig. 3). As the primary organ for nutrient and water absorption, roots exhibit a higher sensitivity to water than seeds and plumules [29]. In addition to activating enzymatic oxidative systems, plants also eliminate ROS through the synthesis of non-enzymatic antioxidants [30]. Under drought stress, genes involved in alleviating oxidative stress and phenylpropanoid biosynthesis are significantly enriched in the seedlings of *Psammochloa villosa* [13]. Research has found that the expression of *BGLU* family genes positively contributes to the enhanced synthesis of flavonoid antioxidant metabolites [31, 32]. We observed that genes specifically expressed in seeds under FW conditions are enriched in the phenylpropanoid biosynthesis pathway, which produces phenolic compounds that clear ROS (Fig. 7C). In the pathway of biosynthesis of various plant secondary metabolites, seven BGLU family genes involved in the biosynthesis of pentagelloyl glucose and coumarin, which are related to antioxidation, were upregulated under FW conditions (Fig. 7C, D, and E). Additionally, we found higher levels of glutathione under FW conditions (Fig. 3F). Therefore, we hypothesize that seeds clear ROS produced due to active metabolism under well-watered conditions by activating the expression of non-enzymatic clearance systems, while modulating oxidative stress caused by water stress through the synthesis of antioxidant enzymes (Figs. 3 and 7).

### Adequate water supply enhances metabolism within seeds

Starch, as the primary reserve within the seed endosperm, predominantly serves as a carbon source for energy metabolism and the synthesis of metabolic products during seedling growth [15]. The hydrolysis of starch involves multiple enzymatic reactions, among which the high activity of α-amylase and sucrose synthase plays a pivotal role [33]. Our experimental results reveal higher α-amylase and SS activities in seeds compared to radicles and plumules (Fig. 2D and E), indicating active starch hydrolysis within the seeds. The elevated activity of TPS suggests an active synthesis of trehalose-6-phosphate (Tre6P), which acts as a sugar starvation signal to maintain sucrose balance between cells [34]. Consequently, we postulate that a significant amount of sucrose within the seeds is either consumed by active metabolism or transported by sucrose transport proteins to the corresponding organs to meet developmental demands during the seedling phase [33, 35]. Our findings of higher glucose content and lower sucrose content in the seeds are consistent with this hypothesis (Fig. 2B and C). For a period after seed germination, the reserves within the seed endosperm are primarily mobilized to sustain the vigor and growth of the seedling, and enhancing sugar metabolism within the seeds is beneficial for maintaining seedling vitality [17, 36]. Research has shown that high activity and expression of α-amylase enhance the rate of sugar metabolism [33, 35–37]. Our results demonstrate higher α-amylase and SS activities, as well as lower sucrose and glucose contents in seeds treated with FW conditions (Fig. 2). In conjunction with the upregulation of the *AMY2* gene, which directs starch breakdown, and the upregulation of genes *TPS6*, which maintain sucrose balance, under FW conditions (Fig. S6A and S6B). Pathways related to energy metabolism, such as valine, leucine, and isoleucine degradation, the citrate cycle (TCA cycle), oxidative phosphorylation, and glyoxylate and dicarboxylate metabolism (Fig. S5), are highly enriched. Our data suggest that the heightened metabolism in seeds under conditions of adequate water supply promotes seedling growth.

### ABA content inhibits sugar metabolism

The expression of α-amylase genes is primarily induced by GA signals and sugar demand, mediated through the GA response complex (GARC) and the sugar response complex (SRC) [1, 17, 21]. Our experiments detected no significant differences in GA levels within the plumules, radicles, and seeds between the two moisture conditions. However, ABA content was consistently lower in the FW treatment by more than 71.52% compared to the SW treatment (Fig. 4B and D). We speculate that the inhibited growth of plumules and radicles under SW conditions may be orchestrated by ABA and sugar accumulation signals. Studies have shown that key components of ABA, such as *ABI4* and *ABI5*, maintain the seed embryo in a dormant state, thereby inhibiting seedling establishment [38]. Sugar accumulation signals have been confirmed to suppress the expression and synthesis of α-amylase mRNA and protein in rice (*Oryza sativa*) and barley (*Hordeum vulgare*) [39]. Our results indicate a higher accumulation of ABA within the seeds under SW conditions, with the upregulation of related genes *ABF4*, *BZIP46*, and *SAPK3* under SW conditions compared to FW conditions (Fig. 4D and S6D). The sucrose and glucose content within the seeds was 157.30% and 64.58% higher under SW conditions than under FW conditions, respectively, aligning with the phenomenon of sugar accumulation (Fig. 2A and B, and 2E). The interactive suppression of α-amylase expression by ABA and sugar accumulation signals leads to lower α-amylase activity under SW conditions [21], thereby reducing sugar metabolism within the seeds.

## Conclusions

Our experiments demonstrate that adequate water supply significantly enhances the germination rate of *F. kryloviana* seeds and the emergence rate of seedlings, as well as promotes the growth of both roots and shoots in the seedlings. Under conditions of ample moisture, the notable increase in α-amylase activity within the seeds and the activation of sugar metabolic pathways underscore the pivotal role of water in regulating seed energy metabolism. Furthermore, the upregulation of genes related to the antioxidant system within the seeds, as revealed by transcriptomic analysis under well-watered conditions, indicates that plants employ an enhanced antioxidant defense mechanism to counteract potential oxidative stress. In summary, our findings underscore the importance of water management during the seedling stage for the cultivation of *F. kryloviana* in arid regions, such as the Qinghai-Tibet Plateau. The results of our experiments also hold significant reference value for guiding the cultivation and management of other crops in similar arid environments. Future research should further explore the regulatory networks of plant growth under water stress and investigate how genetic improvement or agronomic practices can enhance plant water use efficiency and stress resistance.

## Materials and methods

### Soil characteristics investigation

At local area, we identified 3 soil sampling sites for our investigation. The soil moisture content, as determined by the average weight of water in the soil samples collected from these sites. Sample point 1 represents the location of the planting area of *F. kryloviana*, while sample points 2 and 3 are approximately 5 km away from sample point 1. In June, the month of sowing, soil samples were taken for soil testing. For each sample point, soil samples were collected from three layers: 0–10 cm, 10–20 cm and 20–30 cm. Soil sampling operations at each sample point refer to Rawlins [40]. To obtain representative soil samples, five incremental soil samples were collected using a Dutch auger at the corners and centre of a square with a side of length 10 m and combined to form a composite sample. The soil samples were then analyzed for weight moisture content.

Follow local customs and consider the planting depth for *F. kryloviana* seeds were typically 5–10 cm, subsequent moisture content settings were based on the moisture content (11.61%) of the 0–10 cm soil layer (Fig. S1).

### Germination rate, emergence rate, and seedling growth

Our experimental material is *Festuca kryloviana cv*. Huanhu. The seeds were sourced from the Qinghai University Academy of Animal Science and Veterinary Medicine. Germination tests were conducted based on the soil moisture content surveyed in the field. We established a moisture gradient in the germination box by placing three layers of filter paper at the bottom, creating moisture levels of 0%, 5%, 10%, 15%, and 20% (with water accumulation at the 20% moisture level). The incubator conditions were maintained at a relative air humidity of 50%, a temperature of 20 °C, a light intensity of 30 µmol m^− 2^ s^− 1^, and a photoperiod of 12 h (light/ dark). Unless otherwise specified, the same parameters were maintained for subsequent experiments. Each treatment consisted of three replicates, each containing 50 seeds.

In the germination box, germinated seeds (radicles approximately half the length of the seeds) were transferred to soil for seedling growth experiments. The sowing depth for the post-germinated seeds was maintained at 5 cm. We set soil moisture levels at 0%, 5%, 10%, 15%, 20%, 25%, and 30% (with significant water accumulation at 25-30% soil moisture) to observe the emergence rate of seedlings. Building upon the observed differences in seedling emergence rates across various moisture conditions (Fig. 1B), we further designed growth and metabolic assays for seedling organs under contrasting hydration levels. To assess seedling growth under varying soil moisture conditions, we simulated two moisture levels— one closely matching the soil moisture content during the background survey (stress water,10%, SW) and the other approximating the soil moisture content at the optimal emergence rate (full water, 20%, FW) (Fig. 1B) in the germination box. Under these two moisture conditions, we analyzed various phenotypic traits, physiological characteristics, and gene expression indicators in the seedlings. We experimental design concept in Fig. S8.

### Phenotypic traits and physiological characteristics of seedlings

The day on which the seeds were transferred post-germination was designated as day 0. We recorded the lengths of radicles and plumules for each treatment every two days. On the 8th day of the experiment, we observed variations in the lengths of both radicles and plumules (Table 1). To ascertain the physiological status of the organs (seed, radicle and plumule), sampling was conducted on the eighth day, with a portion of the samples utilized for physiological assays and another portion reserved for RNA sequencing (RNA-Seq) analysis. For the sugar metabolism of the organs, we quantified the activities of starch, sucrose, glucose, trehalose-6-phosphate synthase (TPS), sucrose synthase (SS), and α-amylase in the radicles, plumules, and seeds. Our analytical approach employed a kit-based method (Ke Wei Luo Company, Tianjin). To facilitate the differentiation of samples derived from various organs in our subsequent studies, we have assigned abbreviated nomenclature to each organ sample. Specifically, the acronyms CKS, SWS, FWS, SWL, and FWL are utilized to represent the following: CKS refers to seeds at the stage of just-germinated; SWS refers to seeds grown for eight days under SW treatment; FWS refers to seeds grown for eight days under FW treatment; SWL refers to plumules grown for eight days under SW treatment; and FWL refers to plumules grown for eight days under FW treatment.

#### Hormone and antioxidant index determination

The methods for determining the contents of zeatin (ZR), GAs, auxins (IAA), and ABA in each organ are referenced from Pan [41]. The methods for determining the contents of MDA, POD, SOD, CAT, GSH, and ascorbic acid are referenced from Pravisya [9].

### RNA sampling and extraction

The RNA sample preparation method is referenced from Zhang [3]. RNA samples were collected on the 0th and 8th days after noting the seedling measurements. On day 1, seeds were sampled with three biological replicates. On day 8, the seed and plumule of the seedling were sampled with three biological replicates. The 15 samples were immediately frozen in liquid nitrogen for RNA extraction. Total RNA was extracted from the plant materials using TRIzol Reagent (Life Technologies, USA) according to the manufacturer’s instructions. The concentration and purity of the total RNA were checked using a NanoDrop spectrophotometer (TermoFisher Scientifc) and an Agilent 2100 Bioanalyzer (Agilent Technologies, USA), respectively.

#### RNA sequencing and de novo assembly

Fifteen cDNA libraries were constructed from cDNA prepared from seedlings using the Illumina TruSeq RNA Sample Preparation v2 kits according to the manufacturer’s instructions (Illumina Inc., San Diego, USA). The libraries were sequenced on the Illumina HiSeq 2000 instrument by GENE DENOVO (Guangzhou, China) using paired-end sequencing technology, with the length of paired-end reads being 90. Raw reads produced from the sequencing were scrutinized for quality in terms of total raw reads, total clean reads, Q20% (proportion of nucleotides with quality value > 20), N percentage (proportion of unknown nucleotides in clean reads), and GC percentage. Method referenced from Hu [42]. The RNA-Seq raw reads were processed to obtain high-quality reads by removing the adapter sequences and low-quality bases at the 3’ end, trimming low-quality bases (Q < 20) from the 5’ and 3’ ends of the remaining reads. Reads filtering out reads containing ‘N’ and greater than 10 bp were considered for analysis. Clean reads were assembled into contigs, transcripts and unigenes with Trinity software (http://trinityrnaseq.sf.net). RPKM was used to normalize the abundances of transcripts [43]. More than a 2-fold change was used to identify the significance of different gene expression between different treatment lines.

#### Bioinformatics analyses

Thereafter, four functional databases (KOG, KEGG, NR, and SwissProt) were utilized for annotation analysis. The expression levels for all transcripts were quantified by calculating FPKM (fragments per kilobase of transcript per million mapped reads) [44]. Differentially expressed genes (DEGs) were screened with a threshold of |log2 (fold change) | >1 and a statistical significance of p-value < 0.05 using the NOISeq method [45]. Gene ontology (GO) and KEGG pathway enrichment of DEGs were performed with the Omicsmart analysis platform (https://www.omicsmart.com/).

#### Quantitative (q)RT-PCR validation

After analyzing the RNA-seq data, we selected 14 genes for real-time qRT-PCR from germinated plumules and seeds. The related species *Festuca arundinacea* GAPDH (IGG no. GT035008) was used as the housekeeping gene and to normalize the expression data [46]. The primer sequences are listed in Table S2. The qRT-PCR verification system used 2×SYBR Green Master Mix (Sparkjade, China) with the LightCycler^®^ 96 (Roche, Germany). The qRT-PCR amplification conditions were as follows: 94 °C for 3 min, followed by 40 cycles at 94 °C for 5–10 s and 60 °C for 30 s. All validations were performed in three biological and technical replicates. Relative quantitative data were calculated using the 2^−ΔΔCT^ method.

### Statistical analysis

The data analysis software used SPSS 25.0 (IBM, USA). The turnkey’s method was used for inter-processing analysis of variance and multiple comparisons, and the statistical significance was set at *P* < 0.05. The data were displayed as mean ± standard deviations. Origin 2021 (Origin Lab, USA) was used for drawing.

### Electronic supplementary material

Below is the link to the electronic supplementary material.

Supplementary Material 1

## Data Availability

Sequence data that support the findings of this study have been deposited in the China National Center Bioinformation Genome Sequence Archive (GSA) as accession number PRJCA024968. The datasets used and/or analyzed during the current study are available from the authors on reasonable request (Zhenghai Shi, 173450676@qq.com).
