# Supplementary material for: Adequate water supply enhances seedling growth and metabolism in Festuca kryloviana: insights from physiological and transcriptomic analys
Source: BMC Plant Biol. 2024 Jul 26;24:714. doi: 10.1186/s12870-024-05353-5 (PMC11282697; doi:10.1186/s12870-024-05353-5)
Supplement: Supplementary file 1 — Supplementary Material 1 [file 12870_2024_5353_MOESM1_ESM.docx]

**Fig. S1** Distribution of soil moisture content at depths of 0-30 cm in the investigated area.

**Fig. S2** Physiological correlation analysis of seedling organs. (A) Plumule; (B) Radicle; (C) Seed.


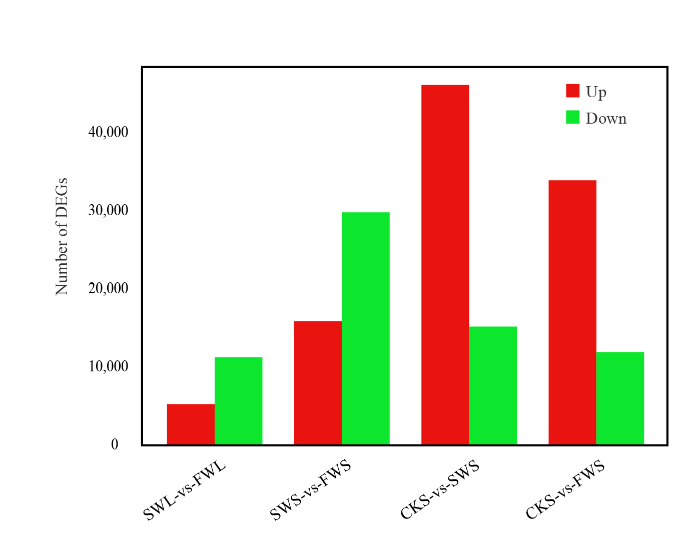


**Fig. S3** Numbers of differentially expressed genes in seeds and plumules during seedling growth. CKS refers to seeds at the stage of just-germinated; SWS refers to seeds grown for eight days under SW treatment; FWS refers to seeds grown for eight days under FW treatment; SWL refers to plumules grown for eight days under SW treatment; and FWL refers to plumules grown for eight days under FW treatment.


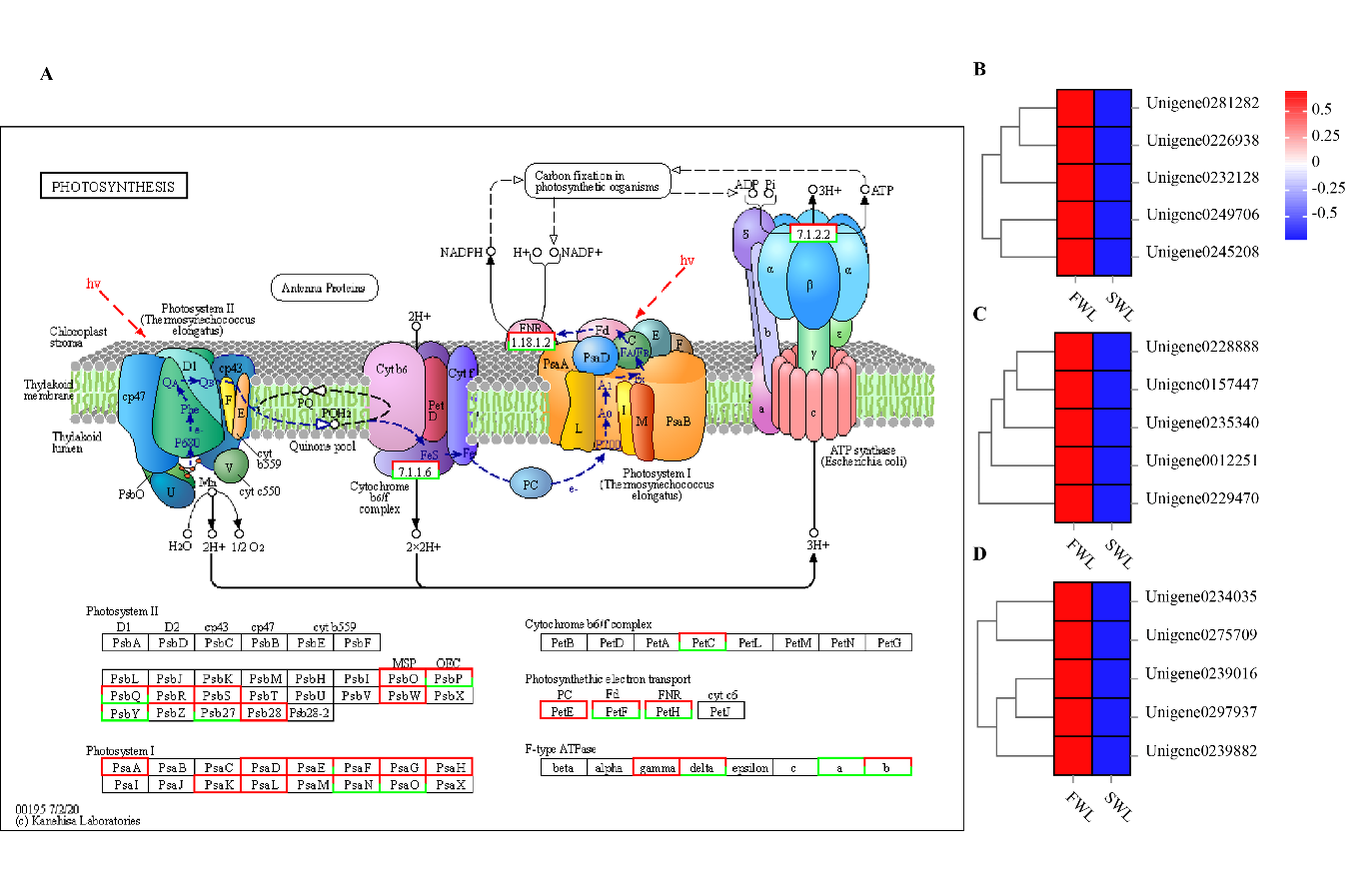


**Fig. S4** Expression of Differential Genes in the Photosynthesis Pathway. (A) Overview of the Photosynthesis pathway; (B) Major differentially expressed genes in Photosystem I; (C) Major differentially expressed genes in Photosystem II; (D) Major differentially expressed genes in Photosynthetic electron transport.


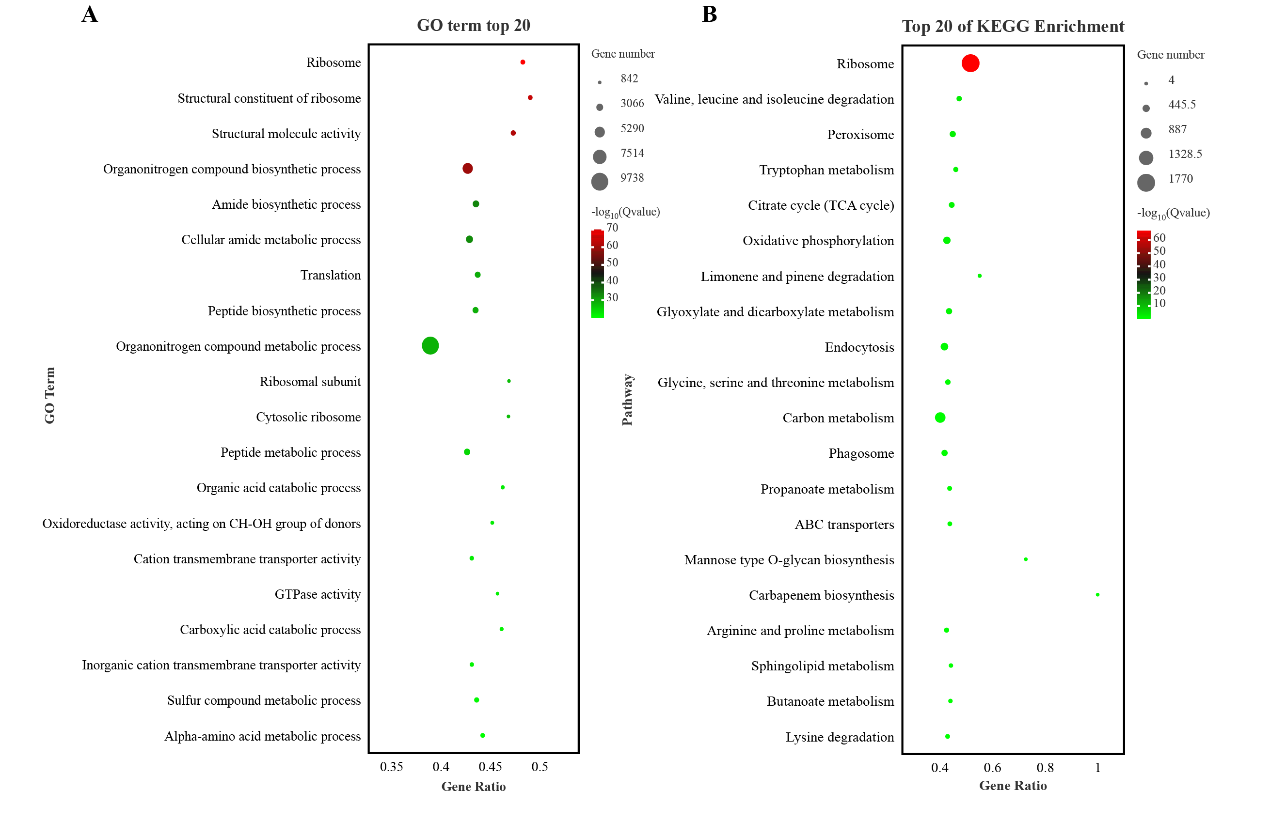


**Fig. S5** GO and KEGG enrichment analysis of seeds in SWS vs FWS groups.


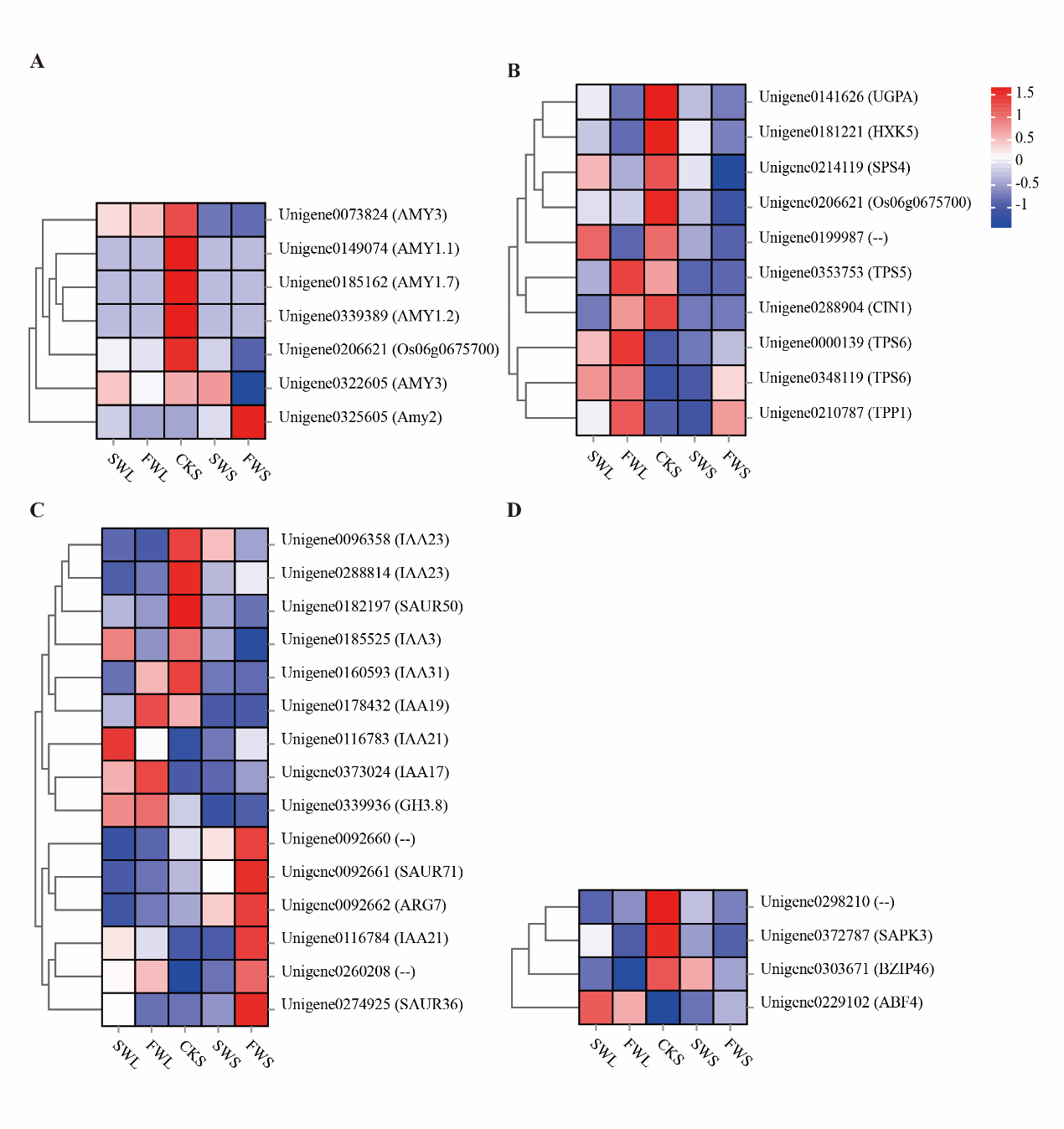


**Fig. S6** Expression of differentially expressed genes involved in sugar metabolism within seeds. (A) Differentially expressed genes in the starch degradation pathway; (B) Differentially expressed genes in sucrose degradation, synthesis, and homeostasis; (C) Genes related to the biosynthesis and signaling of auxins; (D) Genes associated with the biosynthesis and signaling of abscisic acid (ABA).

**
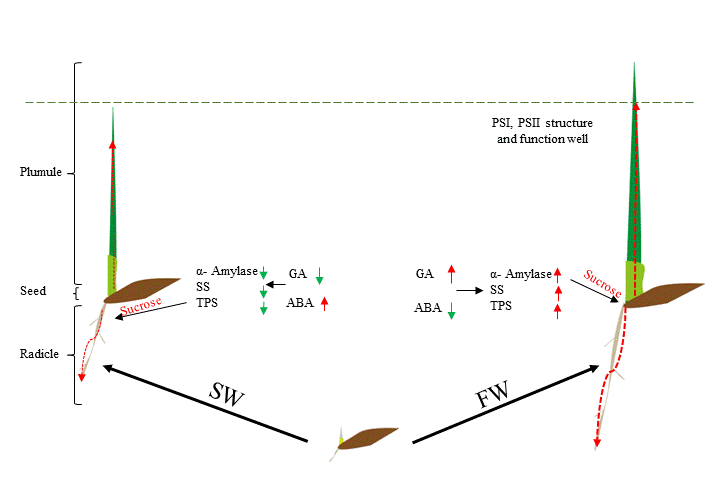
**

**Fig. S7** The main phenotypes and physiological metabolic processes of seedlings under different water conditions.

**
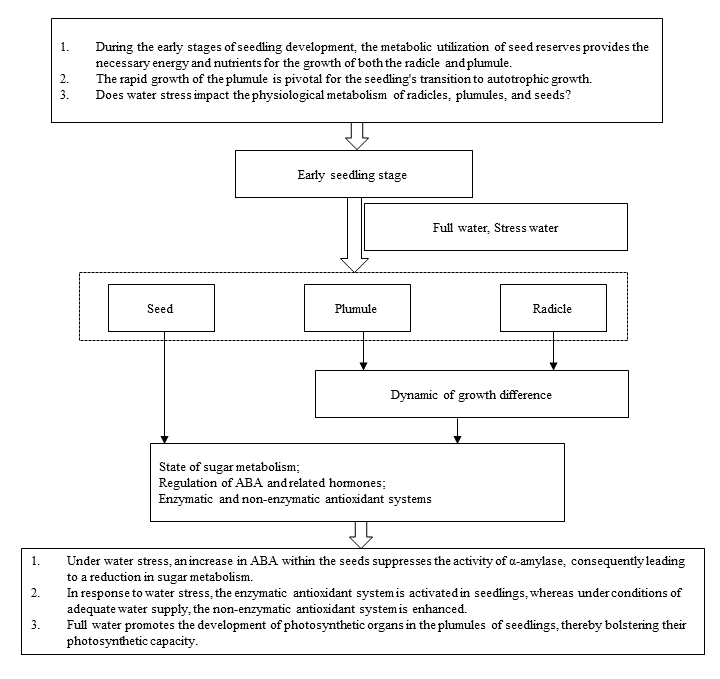
**

**Fig. S8** Our experimental design concept diagram.

**
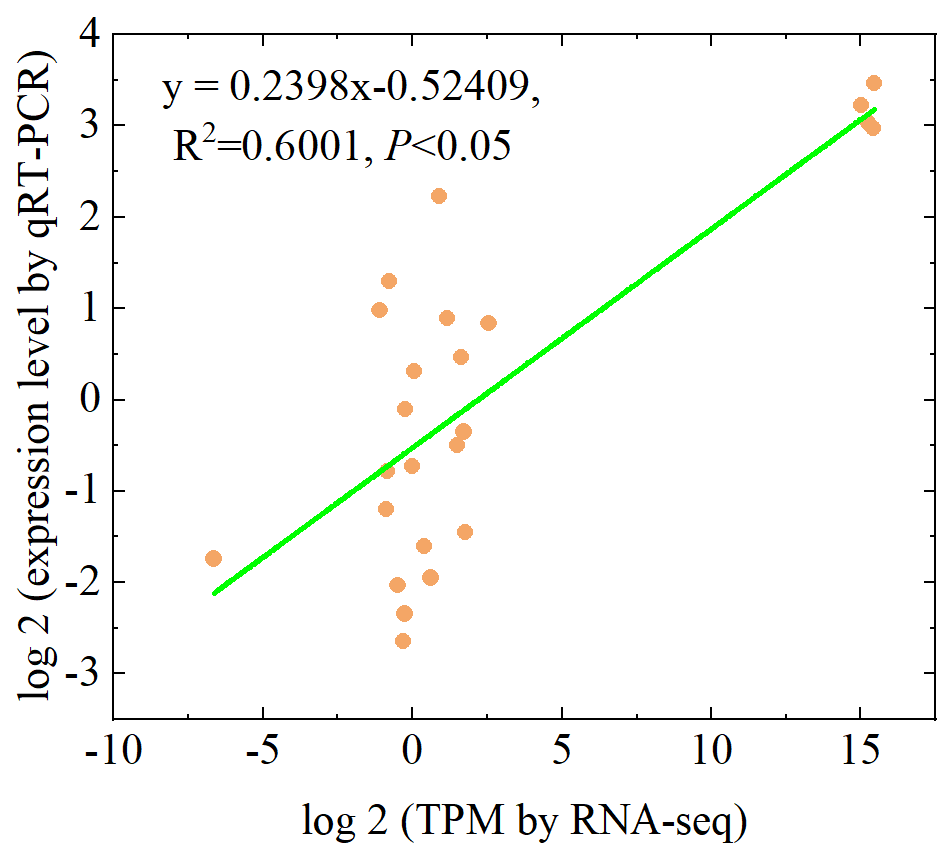
**

**Fig. S9** **Correlation of the RNA-seq and qRT-PCR results.**

**Table S1** Analysis of variance for seedling growth traits. The values represent the *F*-statistics for the independent variables; "**" indicates a highly significant effect. M, D indicates moisture content and days, respectively.

|  | Radicle length | Plumule length |
| --- | --- | --- |
| M | 79.301** | 418.403** |
| D | 308.925** | 280.326** |
| M*D | 5.37** | 6.611** |

**Table S2** Primer sequences used in the manuscript.

| Gene ID | Primer Name | Sequence RTF | Sequence RTR |
| --- | --- | --- | --- |
| GT035008 | GAPDH | TGAGAAGGCAGCCACCTATG | TGCTGTCACCCTGGAAGTCA |
| Unigene0325605 | AMY2 | GACTATGTGAGAACAAGAATCGCTG | GTACGAGTAAACAACTGCACTTCAA |
| Unigene0348119 | TPS6 | GGACATGTTCCAGTTCATCTCCAG | CAACACACCACAGGTCCTCAG |
| Unigene0210787 | TPP1 | GAAAACAACAAATTCTGCGTCTCAG | TTCAGCTTAGGAAAGTCCATCAAGA |
| Unigene0024275 | CAB7 | CAGAAATTGTACACTGTAGATGGGC | GTCGTTGTGTCGGTGAAATATTCTT |
| Unigene0285361 | LHCA4 | GCCTCCTAATAAATGTGGATACCCT | AATCTCCCATTAGCAAGTTCCTTCT |
| Unigene0281445 | CAB13 | CCATGAGTAATGATTTGTGGTACGG | TCAAGTATGAAGGAGTTTGAGCAGA |
| Unigene0252474 | LHCB5 | TCTATTGGACAGATCAGAAATCCCC | TGGATCAGCTCATATGCCTGATATT |
| Unigene0113766 | PER2 | ACCCTGGACAACAAATACTACAAGA | CTTTCACCATTTTCTTCGTCTTGGA |
| Unigene0361196 | CCR1 | CAAGTTCTTCCCACAGTACCCTATC | GTTCGATATCTTGTACGGCTGCTTC |
| Unigene0265738 | ALDH2C4 | GATGTCACTCATGAAGTTCAAGACG | GCGATGTTCAAATCCTTGGTGATAA |
| Unigene0229102 | ABF4 | TTATACGATGGAGTTGGAAGCTGAA | TTACCTCGTTTTTCTGCATTTCCAG |
| Unigene0303671 | BZIP46 | AAGATGGAGGATGATGATTTCTCGT | CCTCCTCTCGACCACCTTCT |
| Unigene0116784 | IAA21 | GCTTGACTCTTCTGCAAGTGTTATT | ATTTTCCATTCGATTCACCATGACC |
| Unigene0096358 | IAA23 | ATGGAAGATGTTCGTTGAGTCCTG | TTGAGAAGATGGGCTTGGAGCTAAG |

**Table S3** qRT-PCR analysis of genes. "/" indicates that no data has been collected.

| Organ | Gene ID | Function | Log (Fold change,2) | |
| --- | --- | --- | --- | --- |
|  |  |  | qRT-PCR | RNA-seq |
| Plumule | Unigene0325605 | AMY2 | 0.878553 | -6.63421 |
|  | Unigene0348119 | TPS6 | 0.308786 | 0.07946 |
|  | Unigene0210787 | TPP1 | -1.95333 | 0.619579 |
|  | Unigene0024275 | CAB7 | 1.035429 | 15.24147 |
|  | Unigene0285361 | LHCA4 | 0.690303 | 15.41448 |
|  | Unigene0281445 | CAB13 | 1.226325 | 15.0218 |
|  | Unigene0252474 | LHCB5 | 0.613126 | 15.44962 |
|  | Unigene0113766 | PER2 | 1.276223 | 0.000012 |
|  | Unigene0361196 | CCR1 | -2.63982 | -0.31377 |
|  | Unigene0265738 | ALDH2C4 | 1.217118 | -0.84712 |
|  | Unigene0229102 | ABF4 | -2.34632 | -0.25941 |
|  | Unigene0303671 | BZIP46 | -1.19801 | -0.88203 |
|  | Unigene0116784 | IAA21 | -0.10341 | -0.23985 |
|  | Unigene0096358 | IAA23 | -2.7242 | -0.49923 |
| Seed | Unigene0325605 | AMY2 | 0.174359 | 2.555384 |
|  | Unigene0348119 | TPS6 | 0.210972 | 1.169703 |
|  | Unigene0210787 | TPP1 | -1.16994 | 1.488609 |
|  | Unigene0024275 | CAB7 | / | / |
|  | Unigene0285361 | LHCA4 | / | / |
|  | Unigene0281445 | CAB13 | / | / |
|  | Unigene0252474 | LHCB5 | / | / |
|  | Unigene0113766 | PER2 | 0.127294 | 1.625384 |
|  | Unigene0361196 | CCR1 | 1.229214 | 0.886745 |
|  | Unigene0265738 | ALDH2C4 | 0.062988 | 1.77388 |
|  | Unigene0229102 | ABF4 | -1.60729 | 0.402131 |
|  | Unigene0303671 | BZIP46 | 1.292589 | -0.78067 |
|  | Unigene0116784 | IAA21 | -0.35093 | 1.719741 |
|  | Unigene0096358 | IAA23 | 0.97862 | -1.0913 |
